# Cord blood anti-Müllerian hormone levels are higher in female newborns from women with polycystic ovary syndrome (PCOS) when compared to non-PCOS controls, irrespective of body mass index; a prospective case-control study

**DOI:** 10.5935/1518-0557.20230005

**Published:** 2023

**Authors:** Şahin Kaan Baydemir, Yavuz Emre Sukur, Nur Ersan, Ozlem Dogan, Cem Somer Atabekoglu

**Affiliations:** 1 Ankara University School of Medicine, Department of Obstetrics and Gynecology, Ankara, Turkey; 2 Ankara University School of Medicine, Department of Biochemistry, Ankara, Turkey

**Keywords:** anti-Müllerian hormone, cord blood, intrauterine, newborn, obesity, polycystic ovary syndrome

## Abstract

**Objective:**

To compare cord-blood anti-Müllerian hormone levels between female newborns from women with and without polycystic ovary syndrome.

**Methods:**

A prospective case-control study was conducted in Ankara University School of Medicine, Department of Obstetrics and Gynecology between June 2020 and January 2021. In total, 408 women gave birth to a female during the study period. Of those, 45 had a polycystic ovary syndrome-like history. We did not find the preconceptional history of 16 women. Two women were excluded due to other endocrine disorders. The polycystic ovary syndrome group consisted of 27 women with polycystic ovary syndrome that gave birth to a female newborn during the study period and the non-polycystic ovary syndrome control group consisted of 33 women who had regular cycles prior to pregnancy, were never diagnosed with polycystic ovary syndrome, and gave birth to female newborns. The primary outcome measure was the cord-blood anti-Müllerian hormone levels.

**Results:**

The median cord-blood anti-Müllerian hormone levels of female newborns from polycystic ovary syndrome patients were significantly higher than those in the non-polycystic ovary syndrome group (0.33ng/ml *vs*. 0.12ng/ml, respectively; *p*<0.001). In addition, cord blood anti-Müllerian hormone levels were significantly higher in both obese and non-obese polycystic ovary syndrome patients when compared to body-mass-index-matched non- polycystic ovary syndrome patients (0.37ng/ml *vs*. 0.06ng/ml, respectively; *p*=0.013 and 0.30ng/ml *vs*. 0.11 ng/ml, respectively; *p*=0.003).

**Conclusions:**

The cord blood anti-Müllerian hormone levels were higher in female newborns of women with polycystic ovary syndrome when compared to non-polycystic ovary syndrome controls. The effect of polycystic ovary syndrome seems to be greater than body mass index on cord blood anti-Müllerian hormone levels.

## INTRODUCTION

Polycystic ovary syndrome (PCOS) is an endocrine disorder that may present with at least two of the three findings of hyperandrogenism, polycystic ovaries and oli-go/amenorrhea. The prevalence of PCOS varies between 5 and 13%, according to the diagnostic criteria used (Bozdag *et al*., 2016). Its prevalence is higher in women with an-ovulatory infertility, obese women and women with first degree relatives with PCOS (Kahsar-Miller *et al*., 2001; Alvarez-Blasco *et al*., 2006; Azziz *et al*., 2016). The serum anti-Müllerian hormone (AMH) levels, Ferriman-Gallwey scores and ovarian volumes were found to be higher in adolescent girls born to women with PCOS than in adolescent girls born to women without PCOS (Crisosto *et al*., 2019). In addition, serum luteinizing hormone and androgen levels were higher in these girls (Crisosto *et al*., 2019).

Anti-Müllerian hormone, a member of the transforming growth factor β family, is significantly higher in women with PCOS than in the normal population and may play role in the pathogenesis of PCOS by increasing LH secretion (Pellatt *et al*., 2007). In male fetuses, AMH is released from Sertoli cells, and it inhibits the development of Müllerian ducts (Josso *et al*., 2012). In female fetuses, the absence of AMH and testosterone leads to the development of Müllerian ducts and regression of Wolffian ducts (Parker *et al*., 1999). Serum AMH levels are also higher in pregnant women with PCOS when compared to non-PCOS controls. Recently, it has been hypothesized that serum AMH levels may be high in female newborns from mothers with PCOS and elevated AMH *in utero* can lead to PCOS-like conditions (Detti *et al*., 2019; Tadaion Far *et al*., 2019). A recent meta-analysis suggested that female newborns of obese women as well as women with PCOS have higher AMH levels (Zhou *et al*., 2022). On the other hand, male fetuses have already higher levels of AMH than female fetuses (Kollmann *et al*., 2019). Higher AMH levels may indicate increased ovarian reserve or hyperactivation of Sertoli cells (Tadaion Far *et al*., 2019). As expected, high levels in male fetuses inhibit Müllerian duct development, and several Müllerian duct abnormalities may occur in the presence of AMH in female fetuses (Detti *et al*., 2017; 2019; Detti, 2014; Vanky & Carlsen, 2012). High AMH levels exposed during intrauterine period may increase the risk of abnormalities such as non-complex subseptate and bicornuate uterus by delaying the fusion of the Müllerian ducts and preventing reabsorption of midline structures (Detti *et al*., 2017). If the AMH level is found to be high in the new-borns of mothers with PCOS, new studies may reveal new findings related to the rate of Müllerian anomaly detection in these newborns at later ages.

A limited number of studies have investigated the AMH levels in newborns of mothers with PCOS and contradictory results have been published (Detti *et al*., 2019). The small increments in males who already have high AMH levels could be negligible. Also, this relatively small increment would not be expected to cause hypothetical congenital abnormality in males. The aim of the present study was to compare the cord-blood AMH levels between female new-borns of women with and without PCOS.

## MATERIALS AND METHODS

The present prospective case-control study was conducted in Ankara University School of Medicine, Department of Obstetrics and Gynecology between June 2020 to January 2021. The study was approved by the Ankara University Ethical Committee for Human Research (approval no.: I3-177-20; date: 26/03/2020). All of the participants were selected from the patients who were followed up in our gynecology and infertility out-patient clinic in the pre-conceptional period. During the study, 408 women gave birth to a female newborn. PCOS history was present in 45 patients. Among those, two patients who had menstrual irregularities due to thyroid dysfunction and hyperprolactinemia were excluded. We excluded 16 patients whose preconceptional history could not be reached. Women who had a diagnosis of PCOS in pre-conceptional period and give live birth to a female newborn during the study period made up the PCOS group (n=27). Women who had regular cycles prior to pregnancy, not diagnosed with PCOS, and give live birth to female newborn, made up the non-PCOS group (n=33). The groups were divided into obese and non-obese subgroups. Obesity is defined as body mass index (BMI) ≥30 kg/m^2^. The inclusion criteria were spontaneous and singleton pregnancy, age 18-40 years and presence of a female fetus. The exclusion criteria were endocrinopathy that may cause menstrual irregularities such as congenital adrenal hyperplasia, Cushing syndrome and hyperprolactinemia, history of chemotherapy or radiotherapy, and presence of a male fetus.

Polycystic ovary syndrome was diagnosed according to the Rotterdam criteria (Wang & Mol, 2017). The main outcome measure was the cord-blood AMH levels. In addition, we compared the demographics of the PCOS and non-PCOS groups. We took a cord-blood sample during birth from all participants for AMH measurement. The samples were taken from the umbilical artery, after clamping the cord, following delivery of the newborn. The samples were centrifuged and the serum stored at –80°C for up to 6 months before all measurements are performed together. AMH was measured by an enzymatically amplified two-site immunoassay (AMH ELISA; Beckman Coulter, Access immunanalyzer, USA). All the procedure steps were taken according to the manufacturer’s recommendations. The lowest detection limit of the assay was 0.02ng/mL. The mean interassay coefficient of variation (CV) was 3.1%, and the mean intraassay CV was 4.0%. A subgroup analysis was also performed for obese patients (BMI ≥30kg/m^2^).

### Statistical Analysis

Data analyzes were performed by using the SPSS Version 21.0 (IBM Corporation, Armonk, NYC, USA). The samples were tested using the Shapiro-Wilk test to determine distribution normality. According to the results, non-parametric tests were preferred. Continuous variables were compared using the Mann-Whitney U test. The categorical variables were compared using the Chi-square test or the Fisher’s exact test, where appropriate. A *p* value of <0.05 was considered statistically significant. The sample size calculation was based on the previous study by Detti *et al*. (2019), which reported median AMH levels of 0.342ng/ ml and 0.101ng/ml in the umbilical arteries of women with and without PCOS, respectively. According to that, 23 patients were required in each group to have a 90% chance of detecting, as significant at the 5% level, an increase in the umbilical artery, AMH level from 0.101ng/ml in the non-PCOS group to 0.342ng/ml in the study group.

## RESULTS

**Figure 1:**
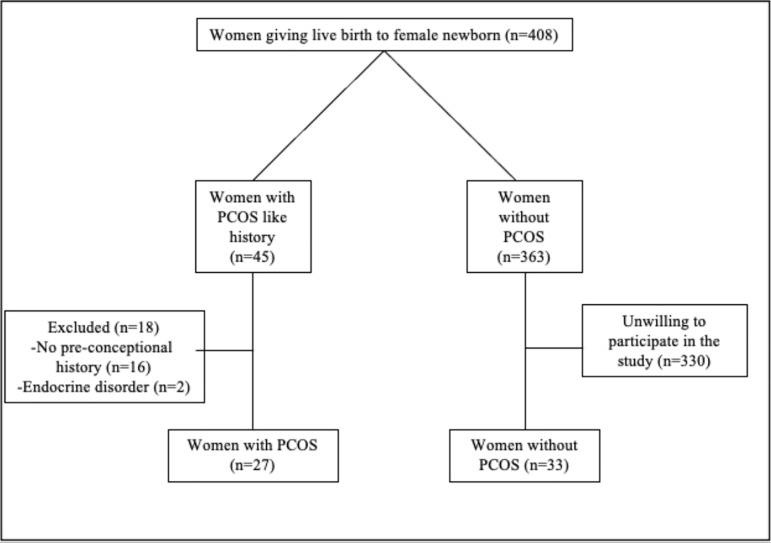
Flow-chart of the study population.

During the study period, 29 women with PCOS gave birth to a female newborn. Among those, two patients who had menstrual irregularities due to thyroid dysfunction and hyperprolactinemia were excluded. As a result, 27 PCOS patients were included in the study group (Figure 1). Thirty-three non-PCOS patients who gave birth to a female newborn were selected as the non-PCOS group. The number of obese patients in the PCOS and non-PCOS groups were 12 (44.4%) and 7 (21.2%), respectively.

The demographic characteristics of the patients were mainly similar between the PCOS and non-PCOS groups (Table 1). The maternal AMH level was significantly higher in the PCOS group (2.5ng/ml *vs*. 1.7ng/ml, respectively; *p*<0.001). Pre-conceptional menstrual irregularities were also significantly higher in the PCOS group when compared to non-PCOS controls (55.6% *vs*. 15.2%, respectively; *p*=0.002) (Table 1).

**Table 1:** The demographic characteristics of the study and control groups.

	PCOS group n=27	Non-PCOS group n=33	*p*-value
Age, years	26 (24.5-29.5)	30 (27-34)	0.203
Parity, n	2 (1-2)	2 (1-2)	0.892
ART pregnancy, n (%)	0 (0)	3 (9.1)	0.244
Menstrual irregularity before pregnancy, n (%)	15 (55.6)	5 (15.2)	0.002
Insulin resistance, n (%)	4 (14.8)	1 (3)	0.100
Gestational age, weeks	39 (38-41)	39 (39-40)	0.210
BMI, kg/m^2^	28 (24-29)	25 (25-30.5)	0.085
Maternal AMH, ng/ml	2.5 (1.3-5.6)	1.7 (1-3.9)	<0.001

Note: PCOS, polycystic ovary syndrome; ART, assisted reproductive technology; BMI, body mass index; AMH, anti-Müllerian hormone. Continuous variables are presented as median (25-75 percentile) and categorical variables are presented as frequency (percentage).

**Figure 2:**
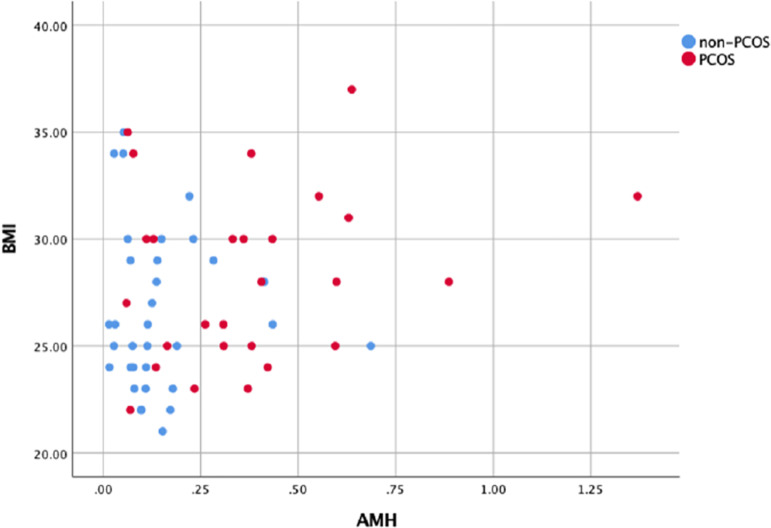
Scatterplot presenting distributions of cord blood AMH levels and BMI of participants in PCOS and non-PCOS groups.

**Figure 3:**
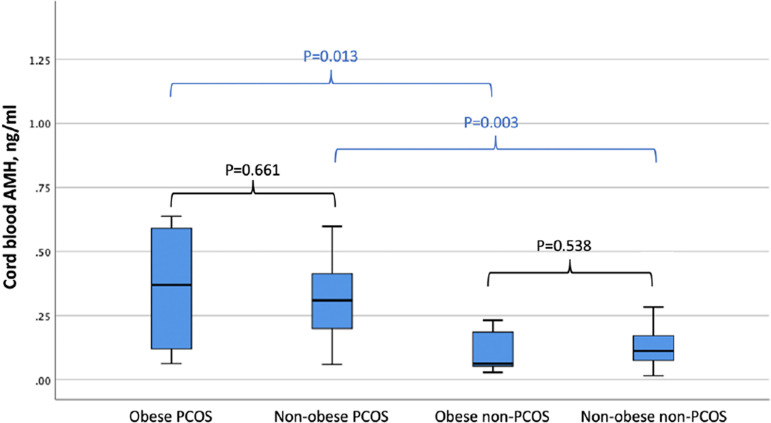
Serum AMH levels in obese and non-obese subgroups.

The median cord-blood AMH levels of female newborns from PCOS patients were significantly higher than those in the non-PCOS group (0.33 ng/ml *vs*. 0.12 ng/ml, respectively; *p*<0.001) (Table 2). In addition, cord blood AMH levels were significantly higher in both obese and non-obese PCOS patients when compared to BMI-matched non-PCOS patients (Table 2). Figure 2 depicts the distribution of PCOS and non-PCOS patients considering BMI and cord blood AMH levels. However, the effect of PCOS was higher than BMI on cord blood AMH levels (Figure 3).

**Table 2:** Comparison of the outcome parameter between the study and control groups.

	PCOS group (n=27)	Non-PCOS group (n=33)	*p*-value
Cord blood AMH, ng/ml	0.33 (0.13-0.57)	0.12 (0.07-0.18)	<0.001
Subgroups according to body mass index - Obese (n=19) - Non-obese (n=41)	0.37 (0.11-0.61) 0.30 (0.16-0.42)	0.06 (0.05-0.22) 0.11 (0.07-0.17)	0.013 0.003

Note: PCOS, polycystic ovary syndrome; AMH, anti-Müllerian hormone. Variables are presented as median (25-75 percentile). Obesity is defined as body mass index ≥30 kg/m^2^.

## DISCUSSION

The present study was conducted to assess the cord-blood AMH levels of female fetuses born to women with and without PCOS. According to the results obtained from this study the cord blood AMH levels of female fetuses born to women with PCOS were significantly higher than those from female fetuses born to women without PCOS. The significant differences also stand in obese and non-obese groups. However, the cord blood AMH levels were similar between obese and non-obese women with PCOS and between obese and non-obese women without PCOS.

Anti-Müllerian hormone levels in pregnant women are lower than those in non-pregnant controls, but AMH levels in pregnant women with PCOS were higher when compared to non-PCOS women (Tata *et al*., 2018). In women with lean PCOS, a significant relationship was demonstrated with hyperandrogenemia and high AMH levels during pregnancy. However, in the same study, this relationship was not found in obese PCOS patients.

AMH can be detected in the cord blood around the 21^st^ gestational week, when the Müllerian ducts complete their development (Vanky & Carlsen, 2012). Tadaion Far *et al*., (2019) suggested that high AMH levels in female newborns to women with PCOS might be due to increased ovarian reserve and granulosa cell proliferation. Some authors believe that the placental transition may also play a role in the occurrence of these high levels. However, there is no evidence to date to show placental transition of AMH hormone (Detti *et al*., 2019; Tata *et al.*, 2018). Whatever the origin, high AMH levels during the intrauterine period may also increase the risk of Müllerian duct abnormalities, such as non-complex subseptate and bicornuate uterus by delaying the fusion of the Müllerian ducts and preventing reabsorption of midline structures (Detti *et al*., 2017). Detti *et al*. (2017) reported that high AMH levels delay the development of Müllerian duct and may cause mild Müllerian abnormalities.

In the present study, we found that the AMH levels in the cord blood of PCOS mothers were significantly higher than in the cord-blood of non-PCOS controls. Detti *et al*. (2019) found that the AMH levels of girls from PCOS mothers were significantly higher than the controls. However, in two recent studies Caanen *et al*. (2016) and Kollmann *et al*. (2019) both failed to show a significant difference in cord-blood AMH levels between female newborns born to PCOS and controls.

We also found that the cord blood AMH levels were significantly higher in both obese and non-obese PCOS patients when compared to BMI matched non-PCOS patients. A recent meta-analysis by Zhou *et al*. (2022), showed that AMH levels were also high in female newborns from obese women, in addition to women with PCOS. Similarly, Tadaion Far *et al*. (2019) divided patients into obese and non-obese subgroups. When obese women were evaluated, they found higher AMH levels in females born from mothers with PCOS than female newborns of mothers without PCOS. However, among non-obese patients, there was no difference in terms of cord blood AMH levels between female newborns of PCOS and non-PCOS women. When all patients were evaluated, AMH levels were found to be higher in newborns from mothers with PCOS (Tadaion Far *et al*., 2019). Our results were partially similar to those by Tadaion Far *et al*. (2019) and Zhou *et al*. (2022), and we additionally found an increased cord blood AMH level in female newborns from non-obese PCOS patients. Our results indicate an increase in cord blood AMH level in PCOS irrespective of BMI.

One of the strengths of our study is the homogeneous patient population. The other one is that there is no age difference between the study groups. Comparison of serum AMH levels in the same age groups provides a more accurate interpretation of the results. The main limitation of our study is the low number of patients included in the study. Another one may be the lack of measuring other hormones such as androgens.

In conclusion, cord blood AMH levels were high among female newborns to women with PCOS when compared to women without PCOS. In addition, the significant difference exists both in obese and normal BMI subgroups. In addition, our results indicate that BMI has no significant impact on cord blood AMH levels either in PCOS or non-PCOS patients. Considering the possibility of Müllerian duct abnormalities due to high AMH levels in the intrauterine period, extensive research should be performed urgently to uncover the exact relationship and possible mechanisms.
